# Preoperative Assessment of the Axilla by Surgeon Performed Ultrasound and Cytology in Patients With Breast Cancer

**DOI:** 10.14740/jocmr2114w

**Published:** 2015-04-08

**Authors:** Gunay Gurleyik, Emin Gurleyik, Ali Aktekin, Fugen Aker

**Affiliations:** a Department of Surgery, Haydarpasa Numune Education and Research Hospital, Istanbul, Turkey; b Department of Surgery, Duzce University Medical Faculty, Duzce, Turkey; c Department of Pathology, Haydarpasa Numune Education and Research Hospital, Istanbul, Turkey

**Keywords:** Breast, Lymph nodes, Metastasis, Ultrasound, Fine needle aspiration

## Abstract

**Background:**

Preoperative evaluation of the axilla, an important prognostic determinant for patients with invasive breast cancer, is achieved by non- or minimally invasive methods to avoid the potential hazards of operative intervention. The aim of this study was to determine statistical power of axillary ultrasound (US) and US-guided fine needle aspiration cytology (FNAC) for evaluating axillary status.

**Methods:**

Axillary lymph nodes were imaged for malignant involvement by high resolution US in 93 breast cancer patients with clinically negative axilla. Cytological samples were obtained by US-guided FNAC from image-suspicious lymph nodes. Cytology-positive patients directly underwent axillary lymph node dissection (ALND). Patients with US and/or cytology-negative axilla underwent sentinel lymph node biopsy (SLNB). Using statistical analysis, US findings and US combined with FNAC were compared with SLNB and final pathology to measure performance.

**Results:**

US was suspicious for metastasis in 38 patients (41%), of whom 16 (42%) were cytology-positive. Axilla was positive in 36/93 patients (38.7%). Sixteen patients with positive FNAC directly underwent ALND. SLNB and/or final pathology was positive in 13/55 patients (23.7%) with negative US (false negative of US) and in 7/22 patients (31.8%) with positive US but negative cytology (false negative of FNAC). SLNB and/or final pathology was negative in 15/38 patients (39.5%) with positive US (false positive of US). Sensitivity, specificity, accuracy, positive predictive value (PPV) and negative predictive value (NPV) of US alone were 63.8%, 73.6%, 69.8%, 60.5% and 76.3%, respectively, and 69.6%,100%, 81.6%, 100% and 68.1%, respectively, for US combined with FNAC.

**Conclusion:**

Statistical measures of the US alone did not achieve a satisfactory value for excluding operative biopsy. US-negative and US-positive but cytology-negative cases still require SLNB for accurate evaluation of axillary status. On the other hand, US-guided positive cytology can obviate SLNB proceeding directly to ALND and avoiding frozen section of sentinel node(s).

## Introduction

The status of a patient’s axillary lymphatic tissue is perhaps the most important factor for predicting breast cancer prognosis. Historically, routine dissection of the axilla was an important component of breast cancer surgery [1]. However, excision of the axillary lymph nodes customarily creates many complications affecting a patient’s quality of life. Presently, an early diagnosis of breast carcinoma precludes the need for axillary lymph node dissection (ALND) in the majority of patients [2, 3].

The concept of sentinel lymph node biopsy (SLNB), which averts the unnecessary excision of lymph nodes in patients with clinically node-negative disease, has emerged as an appropriate method to determine axillary status without formal dissection [1]. Although SLNB is a widely accepted procedure, it carries some disadvantages such as side effects of blue dye, frozen section difficulties, a longer operating time, non-SLN skip metastasis (despite negative SLNB), etc. A positive SLNB indicates axillary dissection. By contrast, more than half of breast cancer cases with positive SLNB have no node involvement beyond SLNs [4].

A non-operative, non- or minimally invasive procedure to determine axillary status before surgery is useful in patients with clinically negative axilla. Non-invasive imaging methods may evaluate axillary lymph nodes for the presence of metastasis. High resolution ultrasound (US), which establishes structural features of lymph nodes and structural changes suggesting malignant involvement, is being increasingly accepted as an appropriate non-invasive method [2, 4-7].

In addition to imaging, fine needle aspiration cytology (FNAC) is a minimally invasive intervention that establishes the cytological features of image-suspicious lymph nodes. We can hypothesize that US and US-guided FNAC would yield important preoperative information about axillary lymph nodes in patients with invasive breast cancer.

In this study, we aimed to determine the effect of US and US-guided FNAC to preoperatively evaluate the axilla, and the possible presence of metastatic lymph nodes requiring ALND without SLNB.

## Materials and Methods

**Figure 1:**
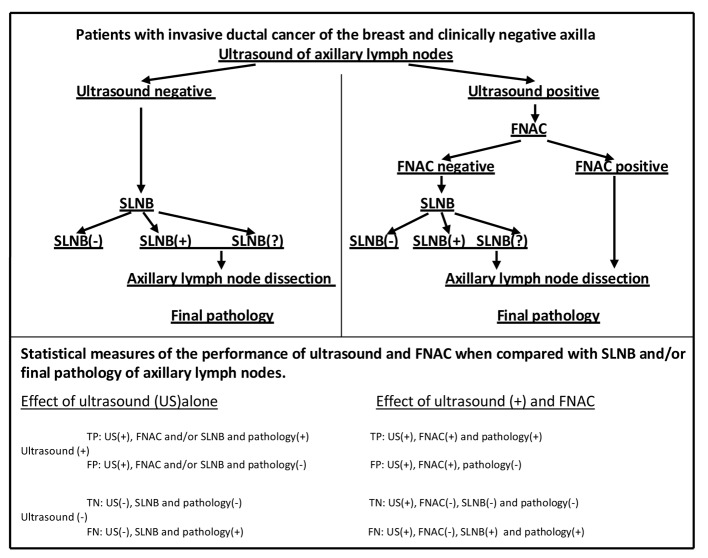
Study protocol. FNAC: fine needle aspiration cytology; SLNB: sentinel lymph node biopsy; SLNB(?): sentinel lymph nodes not found; TP: true positive; FP: false positive; TN: true negative; FN: false negative.

The study was performed on 93 patients with invasive ductal cancer of the breast. All 93 patients had clinically negative axillae with lymph nodes preoperatively assessed by high resolution US and FNAC according to study protocol (Fig. 1).

### Axillary US

Clinically negative axilla of patients with invasive ductal cancer was preoperatively evaluated with high resolution (12 MHz) US (Siemens AG, Munich, Germany). Suspicious lymph nodes were defined by the absence of hilum (loss of hilum fat), cortical thickening > 3 mm, a hypoechoic internal echo and a round shape. Based on these criteria, US suspicious nodes were selected for US-guided FNAC. Patients with US-negative (non-suspicious) axillary nodes underwent SLNB during breast surgery.

### FNAC

Cytological samples were obtained from US suspicious lymph nodes by US-guided aspiration with a 22-gauge needle. Patients with positive cytology (metastatic lymph nodes) proceeded directly to axillary dissection, whereas patients with negative cytology underwent SLNB during breast surgery.

### SLNB

Patients with negative axillary US and patients with positive US but negative cytology underwent SLNB via the patent blue dye method. Blue dye was injected into the peri-areolar region. After 5 min, the axilla was explored for blue stained node(s). Each blue node was dissected and sent to pathology for examination. ALND was performed on patients with positive SLNB with no found SLN.

### Axillary dissection

Level axillary lymph nodes were dissected in three groups of patients: 1) patients with positive US and positive cytology (direct ALND); 2) patients with positive US but negative cytology and with positive SLNB or no found SLN (ALND after SLNB); and 3) patients with negative US but positive SLNB or no found SLN (ALND after SLNB).

### Final pathology

Dissected lymph nodes were histo-pathologically examined for breast cancer metastasis. Using statistical analysis, the results of axillary US and US-guided FNAC were compared with final pathology to determine the relative performance of US and US-guided FNAC.

In analyzing the effectiveness of US and FNAC, performance levels were determined for both US alone and US-associated FNAC. Specificity, sensitivity, accuracy, negative predictive value (NPV) and positive predictive value (PPV) were all calculated in assessing the effectiveness of axillary US and US-guided FNAC.

### The performance of US alone to evaluate axillary status

True positive (TP), false positive (FP), true negative (TN) and false negative (FN) results were determined in calculating specificity, sensitivity, accuracy, NPV and PPV of the US (Fig. 1).

### The performance of FNAC in patients with US-positive lymph nodes to evaluate axillary status

TP, FP, TN and FN results were determined in calculating specificity, sensitivity, accuracy, NPV and PPV of the FNAC in patients with positive US (Fig. 1).

## Results

**Figure 2:**
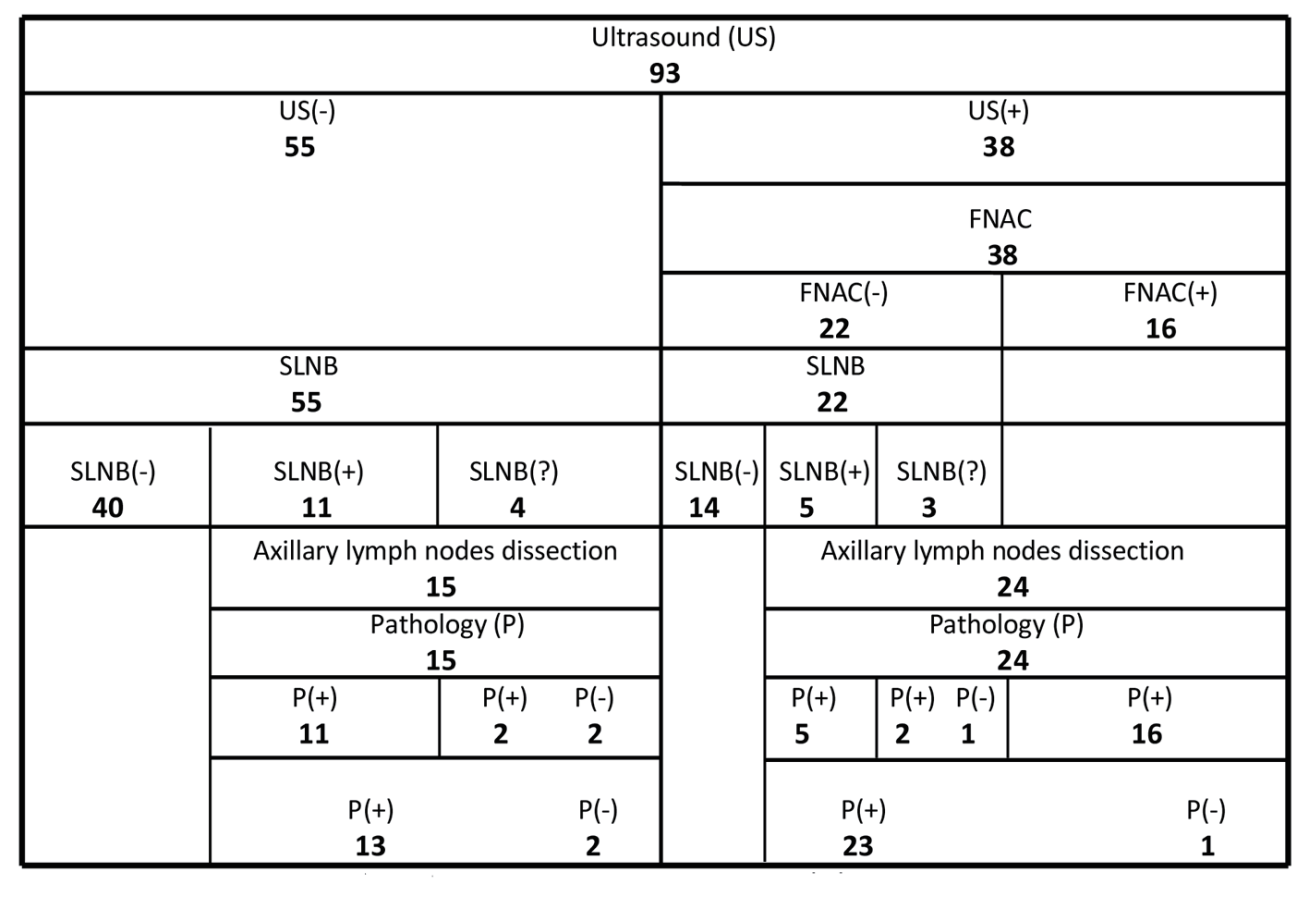
Results of US, FNAC, SLNB and final pathology of patients with breast cancer. FNAC: fine needle aspiration cytology; SLNB: sentinel lymph node biopsy; SLNB(?): sentinel lymph nodes not found.

In this study our cohort of 93 women with invasive ductal cancer of the breast were evaluated by high resolution US. All patients had clinically negative (non-metastatic) axilla with non-palpable lymph nodes. US of the axilla showed abnormal echo-structure of the lymph nodes suspicious for metastasis in 38 patients (41%), and normal structure in 55 (Fig. 2).

In the US-positive group (38 patients), FNAC of the lymph nodes was positive (metastatic) in 16 (42%) of the 38 patients. Axillary lymph nodes were also metastatic per final pathology in all 16 patients with positive FNAC. On the other hand, lymph nodes were metastatic in seven (32%) of the 22 negative FNAC cases after SLNB and final pathology (Fig. 2).

In the US-negative group (55 patients), SLNB of the axilla was positive in 11 of 55 patients (20%). Axillary lymph nodes were metastatic after final pathology in 13 (24%) of the patients with negative US. Lymph nodes were also metastatic in 23 (60.5%) of the patients with positive US. Seventy-seven patients with negative US or positive US but negative FNAC underwent SLNB. Final pathology showed metastatic lymph nodes in 20 (26%) of these 77 patients (Fig. 2).

Level I and II axillary lymph nodes were dissected in 39 patients: directly in 16 patients with positive US and positive cytology; after SLNB in eight patients with positive US but negative cytology; and after SLNB in 15 patients with negative US (Fig. 2).

### Statistical measures of the performance of US alone

US findings and final pathology results and/or SLNB showed that the sensitivity and specificity values of axillary US were 63.8% and 73.6%, respectively (Table 1).

**Table 1:** Statistical Measures of the Performance of Axillary Ultrasound (US) Alone When Compared With Final Pathology

US results (N = 93)	Performance	Definition	Patients	Statistical measures
US(+): 38	True positive	US(+), FNAC(+) or SLNB(+), pathology(+)	23	Sensitivity: 63.8%
	False positive	US(+), FNAC(-) and SLNB(?), pathology(-)	15	Specificity: 73.6%
US(-): 55	True negative	US(-), SLNB(-) or SLNB(?), pathology(-)	42	Accuracy: 69.8%
	False negative	US(-), SLNB(+) or SLNB(?), pathology(+)	13	NPV: 76.3%
				PPV: 60.5%

PPV: positive predictive value; NPV: negative predictive value; FNAC: fine needle aspiration cytology; SLNB: sentinel lymph node biopsy. SLNB(?): sentinel lymph nodes not found.

### Statistical measures of the performance of positive US and US-guided FNAC

FNAC results in patients with positive US and results of final pathology and/or SLNB showed that sensitivity, specificity, PPV and NPV of positive axillary US associated with FNAC were 69.6%, 100%, 100% and 68.1%, respectively (Table 2).

**Table 2:** Statistical Measures of the Performance of FNAC in Patients With Positive Axillary Ultrasound (US) When Compared With Final Pathology

US(+) (N = 38)	Performance	Definition	Patients	Statistical measures
FNAC(+): 16	True positive	FNAC(+), pathology(+)	16	Sensitivity: 69.6%
	False positive	FNAC(+), pathology(-)	0	Specificity: 100%
FNAC(-): 22	True negative	FNAC(-), SLNB(-) or SLNB(?), pathology(-)	15	Accuracy: 81.6%
	False negative	FNAC(-), SLNB(+) or SLNB(?), pathology(+)	7	NPV: 68.1%
				PPV: 100%

PPV: positive predictive value; NPV: negative predictive value; FNAC: fine needle aspiration cytology; SLNB: sentinel lymph node biopsy. SLNB(?): sentinel lymph nodes not found.

## Discussion

Axillary lymph node status is the most important determinant in the regional spread of invasive breast carcinoma. It is, beyond argument, the single most significant prognostic factor in breast cancer [3]. Today, breast cancer cases with clinically negative axilla undergo SLNB to determine axillary lymph node involvement. In cases of positive SLNB or no found SLN, ALND is generally performed. Preoperative evaluation of clinically negative axillae by a non- or minimally invasive method may directly indicate lymph node dissection, thereby avoiding the need for SLNB prefatory to primary breast cancer surgery. High resolution US has gained wide acceptance for preoperative imaging of regional lymph nodes as well as the breast mass itself. We used 12 MHz US for assessing the axillary lymph nodes. Our results proved satisfactory in detecting suspicious nodes. US is helpful for axillary evaluation and lymph node staging [4, 6, 8, 9]. Abnormal echo-structure of lymph nodes may suggest malignant involvement. Grading of nodal involvement on axillary US can be useful for selecting the most suspicious nodes for sampling [7]. Abnormal appearing lymph nodes would likely undergo FNAC with US guidance [10-12]. US-guided FNAC is a highly specific strategy for ascertaining axillary metastases [2, 13]. US-guided aspiration cytology from suspicious nodes may determine the degree of metastasis, along with the need for formal dissection. In this study, we performed both US and US-guided FNAC to avoid SLNB and proceed directly to surgery in breast cancer cases with clinically negative axilla.

Based on statistical measures, which assessed sensitivity and specificity at 63.8% and 73.6%, respectively, the performance of US alone proved unsatisfactory in predicting axillary status for avoidance of SLNB. Previous studies of US performance have reported sensitivity values of 34-71% and specificities of 74-96% [6, 7, 13-17]. For the majority (55/93) of our patients with invasive breast cancer, our axillary US images were not suspicious for metastasis. In 24% of these 55 patients, positive (final) pathology results were discordant due to negative US images. US-negative cases require intraoperative SLNB to prove lymph node involvement. US alone produces a high false negative rate in determining axillary node metastasis. The presence of small metastatic deposits and the extent of nodal metastases were independent factors for false negative US [12, 18]. US showed structurally abnormal lymph nodes in 63.9% of our patients (23 US+/36 pathology+) with positive final pathology. Khout et al [3] reported a rate of 61.2%, and Stachs et al [18] reported a rate of 47.6%. In addition, US-guided FNAC was negative in the majority of our patients (22/38, 58%) with suspicious images. We found a false negative rate for FNAC guided by US of 31.8% (7/22) after final pathology. False negative rates of FNAC have been reported by Diepstraten et al [19] as 25% and by Leenders et al [20] as 31%. Small metastasis size has been reported as the most common cause of false negative results [12, 18]. SLNB is currently the most widely accepted procedure in patients with axillary US negative or US positive but FNAC negative. Positive and no found SLNs indicate formal axillary dissection. Positive (final) pathology results were concordant (23/38) with positive US in 60.5% of our cohort. On the other hand, negative US revealed non-metastatic axillae in the majority (42/55, 76.4%) of our patients. Statistical measures of the performance of US alone, especially NPV and PPV (76.3% and 60.5%), were not persuasive enough to preclude the need for operative intervention to evaluate axillary status. Negative US results do not exclude axillary node metastases with sufficient sensitivity to justify its routine clinical use [16]. In patients with US negative and US positive but FNAC negative, SLNB is the procedure of choice for pathology of lymph nodes. SLNB(+) was performed in 20.8% (16/77) of our patients who had indication of SLN dissection. In Ibrahim-Zada et al’s study [21], the rate was 12.6%.

US suspicious lymph nodes require additional, minimally invasive methods to support positive US results. Aspiration cytology from lymph nodes with suspicious echo-structure is supposed to truly establish the axilla’s pathologic status. In our study, FNAC was positive in 42% of the image suspicious lymph nodes; 33.5% and 60.5% have been reported by two previous studies [5, 22]. Our SLNB and/or final pathology results showed that US alone, with a sensitivity of 69.6% and NPV of 68.2%, was not sufficiently effective to avert SLNB due to negative cytology in patients with positive US. However, final pathology results supported the performance of both positive US and positive FNAC together (specificity 100% and PPV 100%) as a means to preoperatively determine axillary lymph node involvement. Previous reports have confirmed PPV and specificity at 100% for positive FNAC guided by positive US [5, 11, 12, 14, 17, 22]. Statistical measures of US performance in conjunction with aspiration cytology showed an important supportive role for FNAC in confirming US suspicious axilla. In patients who had undergone both positive US and US-guided FNAC, the need for SLNB can be eliminated, with the patient undergoing formal level I and level II axillary dissection during primary surgery. Thirty-six of our 93 patients (39%) had metastatic nodes at final pathology. In 16 of 36 cases (44%), the presence of metastatic nodes was preoperatively determined by US-guided FNAC. In Khout et al’s study [3], 49 of 219 patients (21.5%) had metastatic nodes of which 22 patients (45%) were preoperatively diagnosed by FNAC. US-guided positive FNAC allows patients to be triaged to ALND, thereby avoiding potentially unnecessary SLNB [2]. In other words, preoperative identification of axillary metastases allows the surgeon to proceed directly to ALND [23]. Both US(+) and FNAC(+) were employed in 17% (16/93) of our breast cancer cases who proceeded directly to ALND. This rate represents 16% of the 10,934 patients with breast cancer included in a meta-analysis conducted by Houssami and Turner [2].

Axillary US is a simple, non-invasive and highly effective method for preoperative imaging of lymph nodes in patients with invasive breast cancer. US-guided FNAC from image suspicious lymph nodes is capable of providing significant data. Both US and US-guided FNAC positive results have achieved 100% specificity and PPV. Positive cytological results can potentially obviate SLN dissection. In such cases, surgeon can avoid SLNB and proceed directly to ALND.
